# The role of ferroptosis-related non-coding RNA in liver fibrosis

**DOI:** 10.3389/fcell.2024.1517401

**Published:** 2024-12-09

**Authors:** Guozhu Zhang, Kejia Wu, Xiaobo Jiang, Yuan Gao, Dong Ding, Hao Wang, Chongyuan Yu, Xiaozhong Wang, Naixin Jia, Li Zhu

**Affiliations:** ^1^ Department of Emergency Medicine, The First People’s Hospital of Changzhou, The Third Affiliated Hospital of Soochow University, Changzhou, Jiangsu, China; ^2^ Department of Hepatobiliary and Pancreatic Surgery, The First People’s Hospital of Changzhou, The Third Affiliated Hospital of Soochow University, Changzhou, Jiangsu, China; ^3^ Department of Hepatobiliary and Pancreatic Surgery, The First Affiliated Hospital of Soochow University, Suzhou, Jiangsu, China; ^4^ Kunshan Zhenchuan Community Health Service Center, Kunshan, Jiangsu, China; ^5^ Department of Hepato-Biliary-Pancreatic Surgery, The Institute of Hepatobiliary and Pancreatic Diseases, The Affiliated Changzhou Second People’s Hospital of Nanjing Medical University, Changzhou, Jiangsu, China; ^6^ Department of General Surgery, Wujin Affiliated Hospital of Jiangsu University and the Wujin Clinical College of Xuzhou Medical University, Changzhou, Jiangsu, China; ^7^ Department of Hepatobiliary Surgery, Kunshan First People’s Hospital affiliated to Jiangsu University, Kunshan, Jiangsu, China

**Keywords:** liver fibrosis, ferroptosis, miRNA, lncRNA, circRNA

## Abstract

Liver fibrosis represents a reversible pathophysiological process, caused by chronic inflammation stemming from hepatocyte damage. It delineates the initial stage in the progression of chronic liver disease. This pathological progression is characterized by the excessive accumulation of the extracellular matrix (ECM), which leads to significant structural disruption and ultimately impairs liver function. To date, no specific antifibrotic drugs have been developed, and advanced liver fibrosis remains largely incurable. Liver transplantation remains the sole efficacious intervention for advanced liver fibrosis; nevertheless, it is constrained by exorbitant costs and the risk of postoperative immune rejection, underscoring the imperative for novel therapeutic strategies. Ferroptosis, an emergent form of regulated cell death, has been identified as a pivotal regulatory mechanism in the development of liver fibrosis and is intricately linked with the progression of liver diseases. Recent investigations have elucidated that a diverse array of non-coding RNAs (ncRNAs), including microRNAs, long non-coding RNAs, and circular RNAs, are involved in the ferroptosis pathway, thereby modulating the progression of various diseases, including liver fibrosis. In recent years, the roles of ferroptosis and ferroptosis-related ncRNAs in liver fibrosis have attracted escalating scholarly attention. This paper elucidates the pathophysiology of liver fibrosis, explores the mechanisms underlying ferroptosis, and delineates the involvement of ncRNA-mediated ferroptosis pathways in the pathology of liver fibrosis. It aims to propose novel strategies for the prevention and therapeutic intervention of liver fibrosis.

## 1 Introduction

Liver fibrosis is typified by the persistent accumulation and aberrant distribution of extracellular matrix (ECM) constituents, including type I and III collagen, glycoproteins, and proteoglycans, within hepatic tissue (Zhang et al., 2016). This pathological scar tissue arises from the repeated or continuous damage to parenchymal cells (e.g., hepatocytes or cholangiocytes) induced by factors such as chronic alcohol abuse, non-alcoholic fatty liver disease (NAFLD), and infections with hepatitis B (HBV) and hepatitis C viruses (HCV) (Parola and Pinzani, 2024). Liver fibrosis represents a common pathological development in the progression of various chronic liver diseases. In the absence of effective treatment, it can progress to cirrhosis, hepatocellular carcinoma, and ultimately liver failure, thereby posing a grave threat to human health (Caligiuri et al., 2021). Liver diseases are responsible for approximately two million annual deaths, constituting 4% of global fatalities, with about two-thirds of these mortalities occurring in men due to complications from cirrhosis and hepatocellular carcinoma (HCC) (Devarbhavi et al., 2023). Presently, no definitive cure exists for end-stage liver fibrosis aside from liver transplantation (Ginès et al., 2021). Therefore, the progression of liver fibrosis predominantly determines the quality of life and prognosis of affected individuals. Ferroptosis is a form of iron-dependent programmed cell death characterized by the accumulation of intracellular iron, the buildup of lipid peroxides, and oxidative damage to cell membranes. The concept was initially introduced by Dixon et al., in 2012 (Dixon et al., 2012).

In recent years, advancements in ferroptosis research have elucidated its intimate association with a myriad of diseases. Ferroptosis has been demonstrated to be implicated in the pathogenesis of various diseases, including liver fibrosis formation (Yu et al., 2020), acute kidney injury (Jin et al., 2023), osteoarthritis (Miao et al., 2022), ulcerative colitis (Luo L. et al., 2023), and the apoptosis of cancer cells (Yang et al., 2023). Significant breakthroughs have been achieved across various disease domains, positioning the targeting of ferroptosis as a promising therapeutic strategy for these conditions.

The human genome predominantly produces RNA molecules that do not translate into proteins, commonly classified as non-coding RNAs (ncRNAs). These ncRNAs encompass various types, such as microRNA (miRNA), long non-coding RNA (lncRNA), circular RNA (circRNA), small nucleolar RNA (snoRNA), small nuclear RNA (snRNA), and piwi-interacting RNA (piRNA) (Wang et al., 2017). ncRNAs are implicated in a multitude of physiological and pathological conditions, rendering them a focal point of contemporary research interest. Owing to their diverse molecular functions, ncRNAs have been demonstrated to play pivotal roles in an array of diseases, including cardiovascular and cerebrovascular diseases, rheumatic diseases, and various malignancies (Gournay et al., 2019). Additionally, some researchers propose that ncRNAs act as novel regulators of ferroptosis. The primary mechanisms can be delineated as follows: endogenous sponges of miRNAs, such as LINC00336 and circ-TTBK2, can modulate miRNA expression, influencing the ferroptosis process and ultimately facilitating tumor progression (Wang et al., 2019). Under oxidative stress, autophagy-dependent ferroptosis drives M2 macrophage polarization, inducing the formation of tumor-associated macrophages (TAMs) and consequently promoting tumor progression (Dai et al., 2020). miRNAs such as miR-214 and miR-137 can directly influence the expression of ferroptosis-related genes, while lncRNA can promote ferroptosis through interactions with ferroptosis-associated proteins (Wang et al., 2020).

Targeting ferroptosis-related ncRNAs represents a promising novel therapeutic approach, offering new avenues for disease intervention. Comprehensive research is imperative to elucidate the roles of these ncRNAs in the initiation and progression of ferroptosis. As additional factors associated with ferroptosis are identified in the future, the understanding of how ncRNAs participate in ferroptosis pathways and orchestrate various disease networks will be enhanced. Nevertheless, the mechanisms through which ncRNAs regulate ferroptosis in the context of liver fibrosis remain inadequately elucidated. This paper centers on the mechanisms underlying ferroptosis, recent advancements in ferroptosis-related ncRNAs (microRNA, lncRNA, circRNA), and their interplay with liver fibrosis. Additionally, the potential of targeting ferroptosis-related ncRNAs in hepatic stellate cells (HSCs) or hepatocyte ferroptosis as therapeutic targets is discussed.

## 2 The key cells in liver fibrosis

**FIGURE 1:**
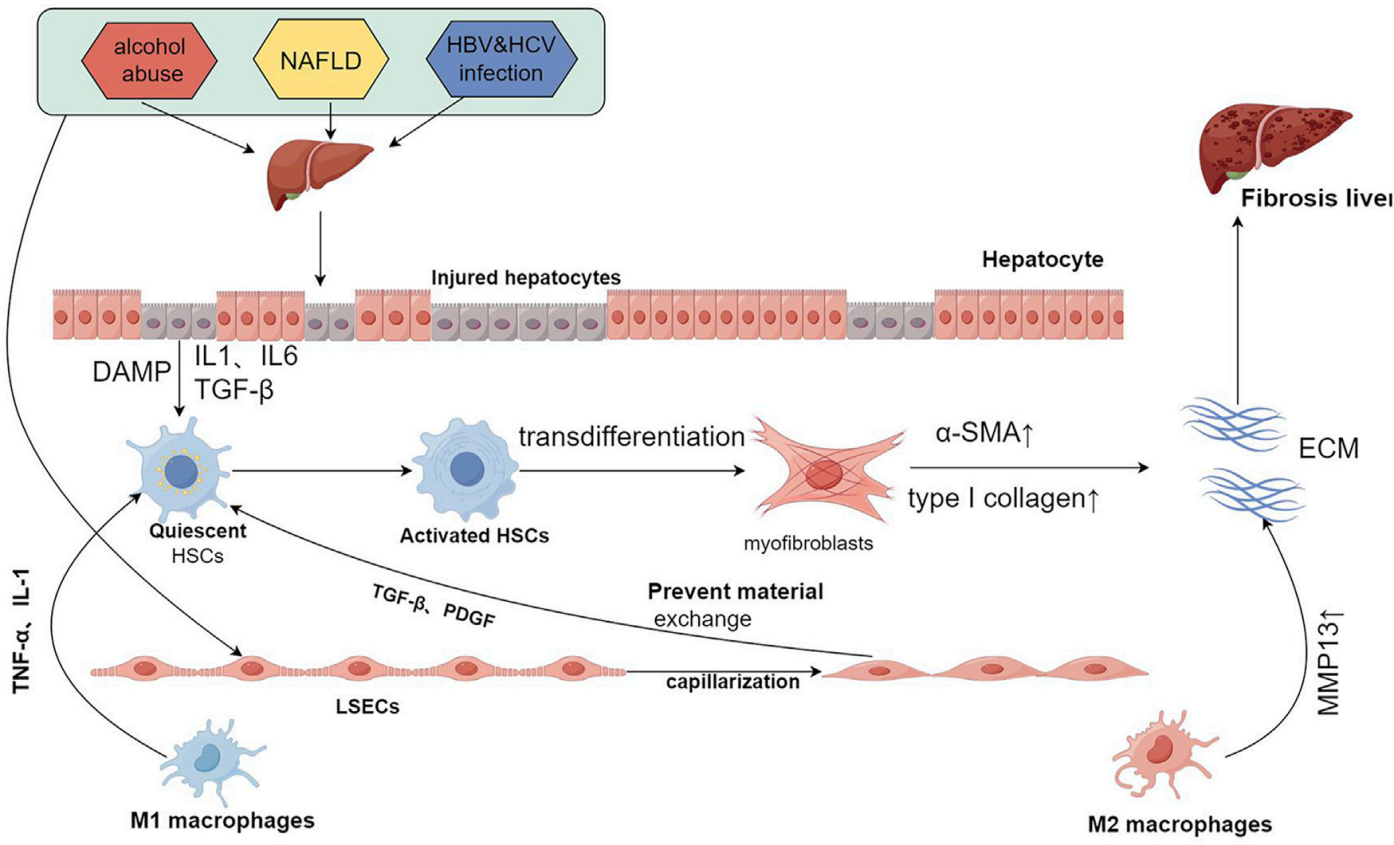
Common mechanisms of liver fibrosis. Repeated injury and persistent assault result in hepatocyte damage, triggering the release of DAMPs, IL1, IL6, TGF-β, and other signaling molecules. This release activates HSCs, which leads to the capillarization of LSECs and the recruitment of immune cells. The activated HSCs undergo continuous stimulation and proliferation, transdifferentiating into myofibroblasts that secrete large quantities of α-SMA and type I collagen, thereby contributing to ECM formation. Following the capillarization of LSECs, material exchange with HSCs is restricted. This condition, coupled with the secretion of cytokines such as TGF-β and PDGF, further stimulates HSCs. Moreover, M1 macrophages secrete TNF-α and IL-1, activating HSCs, while M2 macrophages secrete MMP13, which degrades the ECM. The interactions among these cells create an imbalance between pro-fibrotic and anti-fibrotic mechanisms, and when pro-fibrotic processes predominate, liver fibrosis is ultimately induced. DAMPs:damage-associated patterns; HSCs:hepatic stellate cells; LSECs:liver sinusoidal endothelial cells; α-SMA:α-smooth muscle actin; ECM:extracellular matrix. Created using figdraw.

Liver fibrosis is principally characterized by hepatocyte injury, activation of hepatic stellate cells (HSCs), and substantial extracellular matrix deposition, culminating in fibrotic scar formation that disrupts normal hepatic architecture and impairs liver function (Chen et al., 2024). For a long time, liver fibrosis was considered irreversible, but recent reports suggest that it is a reversible pathological process (Li F. et al., 2023). The mechanisms underlying liver fibrosis are intricate and remain incompletely understood. Regardless of the cause, liver fibrosis involves common molecular mechanisms, including hepatocyte injury and death, immune cell infiltration, capillarization of liver sinusoidal endothelial cells, and HSC activation (Dhar et al., 2020) (Figure 1).

### 2.1 Hepatocytes

Hepatocytes, the principal parenchymal cells in the liver, undertake a plethora of functions under physiological conditions, including metabolism, bile secretion, detoxification, and the synthesis of proteins and lipids (Neshat et al., 2021). Hepatocyte injury and death, caused by various etiological factors, are critical initial events in all liver diseases. Necrotic hepatocytes release damage-associated molecular pattern (DAMP) molecules and inflammatory mediators, which signal the immune system and are pivotal in the progression of fibrosis and inflammation (Gong et al., 2022). Inflammatory mediators incite the activation and proliferation of HSCs. Upon activation, HSCs transdifferentiate into myofibroblasts, which proliferate and secrete extracellular matrix (ECM) proteins, thereby perpetuating the progression of fibrosis (Liedtke et al., 2021). DAMPs released from the mitochondria of injured hepatocytes can directly activate HSCs, thereby accelerating liver fibrosis (An et al., 2020). Studies have shown that silencing lncRNA AK139328 enhances the levels of pAkt and p-eNOS, while reducing hepatocyte death in liver ischemia/reperfusion, thereby demonstrating a protective effect against ischemia/reperfusion injury (IRI) in the liver (Chen et al., 2013). Therefore, therapies aimed at protecting hepatocytes from injury are considered key strategies for treating liver fibrosis.

### 2.2 Hepatic stellate cells

Hepatic stellate cells (HSCs), residing within the space of Disse between hepatocytes and liver sinusoidal endothelial cells (LSECs), undergo aberrant activation that constitutes a principal mechanism in the pathogenesis of liver fibrosis (Liu P. et al., 2022). Under normal physiological conditions, HSCs remain in a quiescent state. However, during liver injury, damaged hepatocytes release components such as DAMPs, which stimulate the transition of quiescent HSCs to activated HSCs. These activated HSCs proliferate and transdifferentiate into proliferative and contractile myofibroblasts. Myofibroblasts secrete type I collagen and α-smooth muscle actin (α-SMA), thereby facilitating the repair of damaged hepatic tissue. The regression of liver fibrosis is associated with the diminution or depletion of HSCs, indicating that deactivating or diminishing HSCs may represent a viable antifibrotic strategy, irrespective of the etiology of liver injury (Tsuchida and Friedman, 2017). Moreover, transforming growth factor-β (TGF-β) is pivotal in the activation of HSCs and is regarded as one of the key profibrogenic cytokines in the liver. Under normal conditions, hepatocytes produce low levels of TGF-β. However, following acute and chronic liver injury, TGF-β secretion significantly increases, activating HSCs to secrete ECM proteins (Dewidar et al., 2019). Chronic injury leads to sustained HSC activation, causing an imbalance between ECM synthesis and degradation, resulting in the excessive accumulation of ECM and the development of liver fibrosis (Roehlen et al., 2020). Reports suggest that TET3, a member of the TET dioxygenase family, promotes TGF-β expression in hepatocytes. Conversely, miRNA let-7 exhibits antifibrotic activity by suppressing TGF-β1 production via TET3 downregulation in hepatocytes (Song et al., 2023).

### 2.3 Liver sinusoidal endothelial cells

Liver sinusoidal endothelial cells (LSECs) serve as a critical barrier between other hepatic cell types and blood, facilitating the exchange of substances between the circulatory system and the space of Disse. In normal hepatic tissue, LSECs display anti-inflammatory, antifibrotic, and immunomodulatory functions, playing a pivotal role in maintaining hepatic homeostasis (Gracia-Sancho et al., 2021). Under normal physiological conditions, LSECs have the capacity to maintain HSC quiescence and support hepatocyte regeneration. However, when LSECs are damaged, they lose this capability, leading to HSC activation, which is critical for fibrosis progression (Koch et al., 2021).During liver injury, LSECs undergo capillarization, leading to diminished levels of vasodilators (e.g., cyclooxygenase-1, nitric oxide) and elevated levels of vasoconstrictors (e.g., endothelin-1, thromboxane A2), severely impairing liver function, activating HSCs, and promoting inflammation and fibrosis (Cheng QN. et al., 2021). Wang et al. identified elevated levels of miR-322/424 in patients with liver cirrhosis. These microRNAs promote fibrosis by targeting Cullin2, thereby enhancing HIF-1α activity to drive angiogenesis (Wang et al., 2021). It has been reported that overexpression of lncRNA Airn sustains LSEC differentiation via the KLF2-eNOS-sGC pathway, thereby preventing capillarization and indirectly suppressing HSCs activation. Furthermore, it boosts the paracrine secretion of HGF by LSECs, facilitating hepatocyte proliferation and mitigating liver fibrosis (Chen et al., 2022). Given the distinctive functions of LSECs, selectively targeting them for liver fibrosis treatment emerges as a promising novel therapeutic strategy.

### 2.4 Macrophage

The liver functions as an immune organ, hosting a diverse array of immune cells from both innate and adaptive immune systems, including neutrophils, lymphocytes, natural killer (NK) cells, and macrophages, each endowed with immunoregulatory capabilities (Xu et al., 2012). Among these, hepatic macrophages are pivotal in initiating inflammatory responses during liver injury, as well as in the development and resolution of liver fibrosis, making them viable targets for antifibrotic therapy (Zhao et al., 2022). Hepatic macrophages are non-parenchymal cells, accounting for 90% of the body’s total macrophages, predominantly composed of resident Kupffer cells (KCs) and macrophages derived from bone marrow monocytes (Cheng D. et al., 2021). Following liver injury or inflammatory stimuli, macrophages undergo polarization, categorized into pro-inflammatory (M1) and anti-inflammatory (M2) macrophages, based on their cytokine profiles (Papadakos et al., 2023). Hepatocytes exposed to intermittent hypoxia release exosomes enriched with miR-421, which suppress the SIRT3/AMPK pathway and subsequently promoting macrophage polarization toward the M1 phenotype (Yang L. et al., 2024). M1 macrophages secrete inflammatory cytokines, including TNF-α and IL-1, which induce the transformation of HSCs into myofibroblasts, thereby contributing to the progression of liver fibrosis. Additionally, inhibiting the Notch signaling pathway to reduce TNF-α secretion by M1 macrophages can alleviate liver fibrosis (Sheng et al., 2020). In a NASH mouse model, treatment with the miR-690 mimic inhibited the function of HSCs and M1 expression, while restoring the number and function of M2 macrophages, thereby attenuating fibrosis progression (Gao et al., 2022). Conversely, M2 macrophages express MMPs, such as MMP13, which degrade ECM and promote the resolution of liver fibrosis (Luo et al., 2019). Hence, liver macrophages exhibit a dual role in liver fibrosis, both promoting its progression and facilitating its resolution.

## 3 Mechanisms of ferroptosis

**FIGURE 2:**
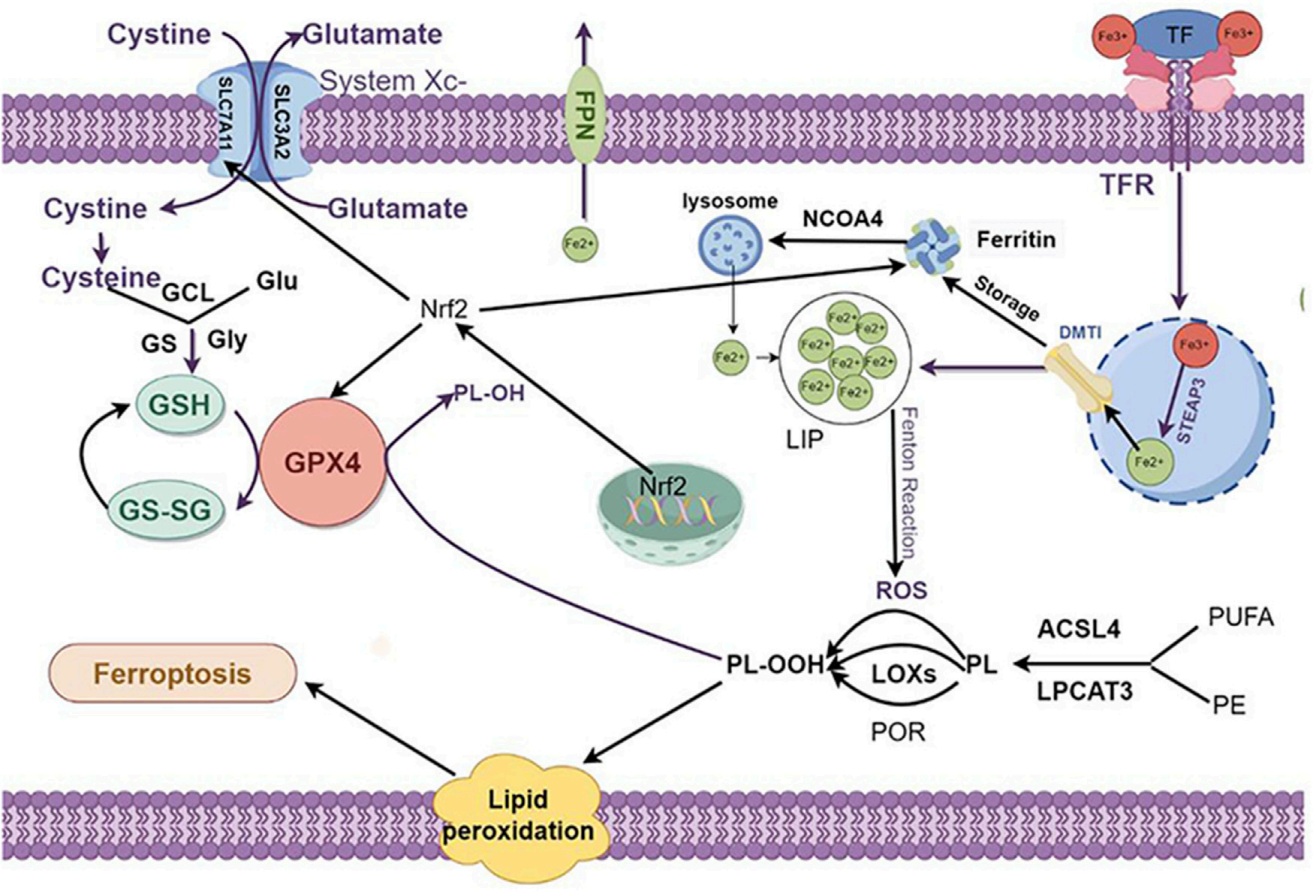
The regulation mechanism underlying cell ferroptosis. Ferroptosis primarily comprises iron metabolism, lipid peroxidation, and amino acid metabolism, with lipid peroxidation at its core. The interplay among these components regulates the onset and progression of ferroptosis. 1. Iron Metabolism: The peroxidation of PL-PUFAs is driven by LIPs and iron-dependent enzymes. After Fe³⁺ binds to TF and is transported into cells via the TFR1, it is reduced to Fe^2^⁺ by the STEAP3, forming an unstable iron pool. Excess Fe^2^⁺ can be exported through FPN or stored in ferritin, which can release Fe^2^⁺ through autophagy mediated by NCOA4. 2. Lipid Metabolism: PL-PUFAs serve as primary substrates for lipid peroxidation. These are catalyzed by ACSL4 and LPCAT3 to form PE and PUFAs. These are then easily peroxidized to PL-OOH, triggering subsequent ferroptotic responses. This peroxidation process is primarily driven by the Fenton reaction or mediated by POR or LOX. 3. Intracellular Antioxidant Systems: System Xc⁻ imports cystine, which is reduced to cysteine. Cysteine, along with Glu and Gly, synthesizes GSH under the sequential actions of GCL and GS. GPX4 mediates the reduction of PL-OOH to PL-OH, and GSSG is regenerated to GSH. Additionally, Nrf2 enhances the expression of SLC7A11 and GPX4 and promotes ferritin synthesis, thereby inhibiting ferroptosis. PL-PUFAs: phospholipid-polyunsaturated fatty acids PL-PUFAs; LIP: labile iron pool; TF: transferrin; TFR1:transferrin receptor 1; STEAP3:sixtransmembrane epithelial antigen of the prostate 3; FPN: ferroportin; NCOA4:nuclear receptor coactivator 4; ACSL4:acyl-CoA synthetase long-chain family member 4; LPCAT3:lysophosphatidylcholine acyltransferase 3; PE: phosphatidylethanolamine; PUFAs: polyunsaturated fatty acids; PL-OOH: phospholipid hydroperoxides; POR: cytochrome P450 oxidoreductase; LOX: lipoxygenases; Glu: glutamate; Gly: glycine; GSH: glutathione; GCL:glutamate-cysteine ligase; GS: glutathione synthetase; GSSG:oxidized glutathione; GPX4:Glutathione peroxidase 4; PL-OH: phospholipid hydroxides; Nrf2:nuclear factor erythroid 2-related factor 2. Created using figdraw.

Ferroptosis exhibits distinct morphological and mechanistic features compared to other cell death modalities, such as apoptosis, necroptosis, necrosis, pyroptosis, autophagy, and cuproptosis (Galluzzi et al., 2018). The hallmark morphological characteristics of ferroptosis encompass reduced mitochondrial size, heightened mitochondrial membrane density, and the disintegration or diminution of mitochondrial cristae and plasma membrane (Liu J. et al., 2022; Wu et al., 2021).In contrast, nuclear morphological changes typical of apoptosis, such as chromatin condensation and apoptotic body formation, are not observed in ferroptosis (Kakarla et al., 2020). These distinctive morphological features are considered hallmarks of ferroptosis. Ferroptosis is primarily dependent on iron ion-mediated oxidative damage. This form of cell death can be triggered when the redox balance between antioxidants and oxidants within the cell is disrupted (Sun et al., 2023). The major regulatory mechanisms of ferroptosis are closely related to iron metabolism, lipid peroxidation, and amino acid metabolism (Stockwell et al., 2017) (Figure 2).

### 3.1 Iron metabolism

**FIGURE 3:**
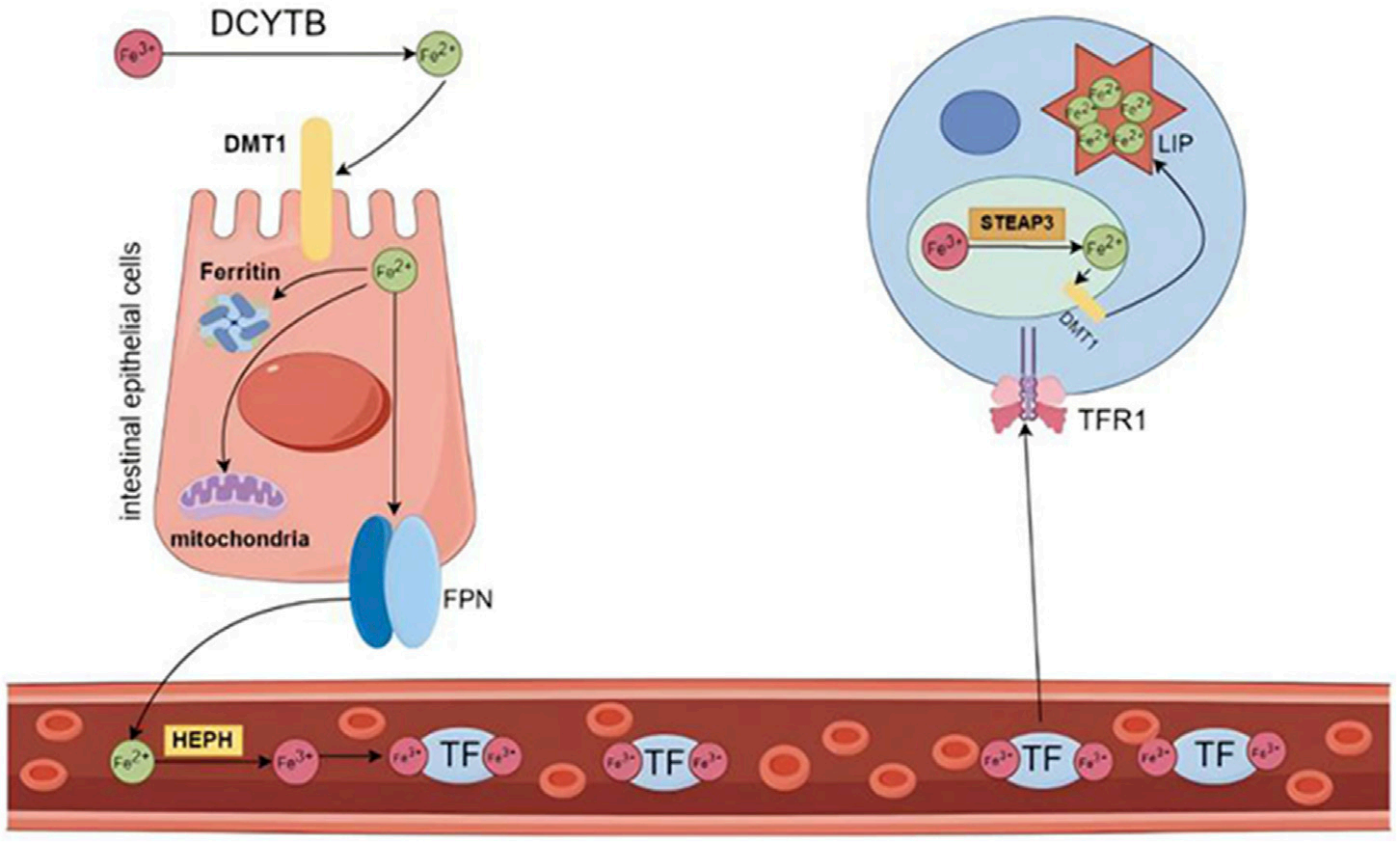
Iron distribution and circulation. Dietary Fe³⁺ is reduced to Fe^2^⁺ by DCYTB and subsequently transported into enterocytes through the apical membrane by DMT1. Once inside the cell, Fe^2^⁺ can be directly utilized for intracellular functions, stored bound to ferritin, or released into the bloodstream. Fe^2^⁺ is exported from the enterocyte by FPN, the only known iron exporter, and is then oxidized by ferroxidase before binding to TF. The majority of circulating iron is bound to TF. Transferrin-bound Fe³⁺ is absorbed into cells via TFR1. Within the endosome, Fe³⁺ is reduced to Fe^2^⁺ by the action of the iron reductase STEAP3 and is subsequently released into the cytoplasm through DMT1, entering the labile iron pool. DCYTB: duodenal cytochrome B; DMT1: divalent metal transporter 1; FPN: ferroportin; TF: transferrin; TFR1: transferrin receptor 1; STEAP3: six transmembrane epithelial antigen of the prostate 3. Created using figdraw.

Iron is a vital trace element integral to human physiology (Ni et al., 2022), playing a pivotal role in fundamental biological processes, encompassing DNA synthesis and repair, mitochondrial respiration, and cellular signaling (Galy et al., 2024). In the body, iron primarily exists in two forms: ferrous ions (Fe^2+^) and ferric ions (Fe^3+^), which play a critical role in cellular functions via redox reactions (Estêvão et al., 2023). Iron metabolism primarily involves the absorption, storage, and utilization of iron (Correnti et al., 2022). In the intestine, dietary iron is reduced to Fe2+ by duodenal cytochrome B (DCYTB) and transported into the cytoplasm of enterocytes through divalent metal transporter 1 (DMT1) (Wang and Babitt, 2019). Within the cytoplasm, ferrous iron can be stored in ferritin or bind to ferroportin (FPN) to cross the basolateral membrane of enterocytes and enter the bloodstream (Pasquadibisceglie et al., 2023). Fe^2+^ is oxidized to Fe^3+^ by ceruloplasmin or hephaestin (HEPH), after which it binds to transferrin (TF), the plasma iron carrier.TF transports Fe³⁺ by engaging with transferrin receptor 1 (TFR1) on the cell membrane, forming a TF-Fe³⁺-TFR1 complex that is internalized via endocytosis, thereby delivering Fe³⁺ into peripheral tissue cells (Bayır et al., 2023). Intracellularly, Fe^3+^ is reduced to Fe^2+^ by the six-transmembrane epithelial antigen of the prostate 3 (STEAP3) ferrireductase. At this juncture, a minor fraction of Fe^2+^ constitutes the labile iron pool (LIP), whereas the majority is sequestered in an inactive form within cytoplasmic ferritin (FT) (Kouroumalis et al., 2023). Nevertheless, the bulk of circulating iron originates not from dietary absorption but from the recycling processes occurring within the body. This recycling predominantly transpires in the spleen and liver, where macrophages phagocytose and degrade iron-rich senescent erythrocytes (Sun et al., 2024) (Figure 3).

The liver functions as a pivotal organ for iron regulation. Hepatocytes produce substantial amounts of ferritin, facilitating excess iron storage and establishing the liver as the body’s primary iron reservoir (Sudarev et al., 2023). Additionally, hepatocytes synthesize and secrete hepcidin, a pivotal hormone in iron metabolism regulation that modulates transferrin activity (Baringer et al., 2023). Elevated hepcidin levels inhibit FPN activity, resulting in iron retention within cells and hindering its mobilization into the bloodstream for utilization by other tissues and organs (Nemeth and Ganz, 2023). In diverse hepatic pathologies, the levels and functionalities of associated proteins, including ferritin, transferrin, and hepcidin, are dysregulated, culminating in intracellular iron accumulation (Hsu et al., 2022).An elevation in the labile iron pool (LIP) is a significant source of intracellular oxidative stress (Wang et al., 2022). Ferrous iron within the LIP participates in the Fenton reaction, catalyzing the generation of hydroxyl radicals. It also partakes in lipid peroxidation reactions, producing lipid radicals such as LOO• and LO•, which attack macromolecules including lipids, proteins, and carbohydrates, leading to oxidative damage and eventual cell death (Gensluckner et al., 2024). Research have shown that miR-485-3p overexpression in HepG2 cell lines suppresses FPN expression and elevates intracellular ferritin levels, leading to the accumulation of intracellular iron (Sangokoya et al., 2013). Zhou et al. identified a significant positive correlation between peripheral blood levels of lncRNA NEAT1 and serum hepcidin in patients with NAFLD. They proposed that lncRNA NEAT1 may regulate hepcidin expression, promoting iron accumulation in hepatocytes and exacerbating cellular injury and death (Zhou and Qiu, 2022).

### 3.2 Lipid metabolism

Lipids are crucial biological macromolecules that play essential roles in cellular life processes as components of cell membranes, organelle membranes (such as mitochondria and the endoplasmic reticulum), and signal transduction pathways (Gerhardtova et al., 2024). Phospholipids are fundamental structures in cell membranes, maintaining cell morphology and coordinating cellular functions. Additionally, phospholipids are highly susceptible to oxidation (Ferreira and Domingues, 2024). The sn-2 position of phospholipids is conjugated with acyl residues, frequently comprising polyunsaturated fatty acids (PUFAs). Due to their multiple double allylic hydrogen atoms, PUFAs exhibit heightened susceptibility to reactive oxygen species (ROS), culminating in the generation of phospholipid hydroperoxides (PLOOH) and free cholesterol. These molecules serve as substrates for lipid peroxidation, a pivotal process in ferroptosis, thereby compromising membrane structure and function (Wu et al., 2021; Zheng and Conrad, 2020).PUFA synthesis is orchestrated by an array of enzymes, notably acyl-CoA synthetase long-chain family member 4 (ACSL4) and lysophosphatidylcholine acyltransferase 3 (LPCAT3), which mediate the incorporation of PUFAs into phosphatidylethanolamine within membrane phospholipids. ACSL4 and LPCAT3 are critical determinants of ferroptosis susceptibility and play a significant role in promoting ferroptosis (Liu J. et al., 2022). In a mouse model of acute myocardial infarction (AMI), myocardial ischemia leads to the accumulation of iron, ROS, and lipid peroxides, supporting the role of ischemia in inducing ferroptosis. Furthermore, miR-450b-5p expression was suppressed in ischemic myocardium. Overexpression of miR-450b-5p downregulated ACSL4 expression and attenuated ferroptosis, whereas miR-450b-5p inhibition produced the opposite effect (Yu et al., 2023). Zhang et al. identified in an acute kidney injury model that miR-124-3p.1 regulates LPCAT3 expression and modulates lipid metabolism, thereby exacerbating ferroptosis and aggravating kidney injury (Zhang H. et al., 2022). Lipid peroxidation can be initiated via non-enzymatic or enzymatic mechanisms. Fe^2^⁺ participate in non-enzymatic Fenton reactions, producing hydroperoxyl radicals (ROO) and hydroxyl radicals (HO). These radicals catalyze the oxidation of PUFAs, initiating lipid peroxidation, thereby intensifying oxidative stress and promoting cell death (Li X. et al., 2023). Lipid peroxidation can also be mediated by lipoxygenases (ALOXs). LOXs, which are non-heme iron-containing enzymes, can directly introduce oxygen into membrane PUFAs and PUFA-containing lipids, triggering peroxidation and ferroptosis (Maheshwari, 2023). Wu et al. identified circPtpn14 as a molecular sponge for miR-351-5p, modulating its expression. In a traumatic brain injury (TBI) model, circPtpn14 was significantly upregulated, resulting in the downregulation of miR-351-5p. Importantly, miR-351-5p suppressed 5-LOX expression. These results indicate that the circPtpn14/miR-351-5p/5-LOX axis enhances 5-LOX expression, facilitating ferroptosis (Wu C. et al., 2022).

### 3.3 Amino acid metabolism

In the human physiological system, several pathways protect against oxidative damage associated with ferroptosis. Ferroptosis can be induced through exogenous and endogenous pathways. The exogenous pathway involves inhibiting cell membrane transport proteins such as cystine/glutamate antiporter (also known as System Xc-, a crucial intracellular antioxidant element) or activating transferrin and lactotransferrin, which induce ferroptosis (Chen et al., 2021). The endogenous pathway involves blocking GPX4, the only intracellular glutathione peroxidase used for lipid peroxidation reduction (Liu et al., 2023). Amino acid metabolism, particularly that of cystine and glutathione, is key to preventing excessive ROS and maintaining normal cellular antioxidant status, and abnormalities in this metabolism are closely related to ferroptosis (Xu et al., 2024).

#### 3.3.1 System Xc-

Glutathione, a thiol-containing tripeptide, is synthesized from intracellular cysteine (Cys), glutamate (Glu), and glycine (Gly) via the sequential catalytic activities of glutamate-cysteine ligase (GCL) and glutathione synthetase (GS) (Fujii et al., 2023). Among these amino acids, Cys is paramount due to its low intracellular abundance, making it the rate-limiting factor in GSH synthesis and a determinant of cellular GSH levels. Intracellular Cys is mainly taken up in its oxidized form, cystine, via the glutamate/cystine antiporter system Xc- from the extracellular environment, where it is rapidly reduced to cysteine (Liu et al., 2020). System Xc- is a component of the heterodimeric amino acid transporter (HAT) family, consisting of two subunits: solute carrier family 7 member 11 (SLC7A11), influencing transporter activity, and solute carrier family 3 member 2 (SLC3A2) (Fantone et al., 2024; Liu MR. et al., 2021). Compounds such as erastin, sulfasalazine, and sorafenib are known to inhibit the System Xc-, thereby affecting the biological processes underlying ferroptosis and demonstrating antitumor effects (Xia et al., 2019). Furthermore, lncRNA DUXAP8 is significantly upregulated in HCC, where it promotes the palmitoylation of SLC7A11, thereby preventing its lysosomal degradation. This ferroptosis-inhibitory mechanism in cancer cells underpins resistance to sorafenib treatment in HCC (Shi et al., 2023).

#### 3.3.2 Glutathione (GSH)/glutathione peroxidase 4 (GPX4)

Mammalian glutathione peroxidases (GPXs) belong to a superfamily of proteins, comprising eight members, each with different structures, biological functions, and expression characteristics, but all capable of reducing hydroperoxides (Flohé et al., 2022). Among these, glutathione peroxidase 4 (GPX4) uniquely acts within the GPX family on intracellular phospholipid hydroperoxides, catalyzing the reduction of toxic PLOOH to non-toxic phospholipid hydroxides (PLOH),thus interrupting the lipid peroxidation reaction (Seibt et al., 2019).GSH is a crucial cofactor for GPX4 in continuously lowering intracellular PLOOH levels, functioning through the interconversion between G-SH and GS-SG, and is vital for the GPX4-catalyzed reaction (Li et al., 2022). Therefore, the cystine transport System Xc- can indirectly affect GPX4 function. Interestingly, ferroptosis can still occur even when intracellular Cys and GSH levels are normal but GPX4 is inactive (Gao et al., 2019). Bi et al. revealed that FUN14 domain containing 1 (FUNDC1), a mitophagy receptor, can bind to GPX4 and recruit it into the mitochondria, where GPX4 induces ferroptosis through mitophagy-mediated liver injury (Bi et al., 2024). miR-137 inhibits mitophagy by downregulating the protein levels of FUNDC1 (Li et al., 2014). Circ0060467 is significantly upregulated in HCC, where it serves as a competing endogenous RNA (ceRNA) that regulates miR-6805. GPX4 is identified as a direct target of miR-6805. Subsequent studies have demonstrated that circ0060467 regulates GPX4 expression by sponging miR-6805 via a ceRNA mechanism, inhibiting ferroptosis in liver cancer cells and facilitating the progression of HCC (Tan et al., 2024). Recent reports indicate that lnc-MRGPRF-6:1 overexpression exacerbates ox-LDL-induced lipid accumulation and promotes lipid peroxidation in macrophages. Mechanistically, it promotes ferroptosis in macrophages by downregulating GPX4 expression, leading to the accumulation of lipid peroxides (You et al., 2023). Consequently, GPX4 plays an essential role in inhibiting lipid peroxidation and is a pivotal mediator in the ferroptosis pathway.

## 4 Ferroptosis-related ncRNA and liver fibrosis

In the past decade, substantial advancements have been made in the development of agonists and antagonists targeting ferroptosis-related proteins, offering promising therapeutic strategies for a range of associated diseases. Sorafenib, a multi-kinase inhibitor employed in advanced HCC treatment, induces ferroptosis in HCC by inhibiting system Xc-. Nevertheless, studies indicate that cancer cells can circumvent sorafenib-induced ferroptosis by activating the Nrf2 antioxidant pathway, resulting in therapeutic resistance (Louandre et al., 2013). Natural active compounds (NAC), including artemisinin, Decursin, Isoliquiritigenin and Wogonoside, alleviate liver fibrosis by modulating iron, lipid, and amino acid metabolism. Nevertheless, the therapeutic potential of certain natural compounds is constrained by low bioavailability and the risk of hepatotoxicity at elevated doses (Wu et al., 2024). Currently, these drugs primarily target key proteins within the core pathways of ferroptosis. However, their modulation of ferroptosis may inadvertently promote drug resistance, perturb normal cellular metabolism, and trigger additional adverse effects. ncRNAs are integral to fundamental biological processes, including growth, development, and metabolism. They achieve this by binding to proteins and participating in the stabilization and regulation of chromatin structure, DNA replication, RNA processing and modification, protein transport, cellular metabolism, development, and proliferation (Nemeth et al., 2024; Zhang Y. et al., 2023). Different ncRNA types exhibit distinct functions and have been shown to play critical roles in modulating ferroptosis (Zhang and Xie, 2024). A range of strategies for ncRNA-based therapies have been proposed, including antisense oligonucleotide (ASO) anti-microRNAs (antimiRs), miRNA mimics, miRNA sponges, therapeutic circular RNAs, and CRISPR-Cas9-based gene editing approaches (Winkle et al., 2021). Considering the heterogeneity in gene expression across individuals, we propose that ncRNA-based therapies present substantial potential for modulating ferroptosis pathways, thereby opening new avenues for personalized liver fibrosis treatment. In this review, we comprehensively summarize and critically analyze the roles of ferroptosis-related ncRNAs in the pathogenesis, progression, and therapeutic strategies for liver fibrosis (Table 1).

**TABLE 1:** The regulatory role of ferroptosis-related ncRNAs in liver fibrosis progression.

ncRNA	Mechanism	Function	Reference
miR-192-5p	Target FNDC3B	Induce ferroptosis in hepatocytes, promote liver injury	You et al. (2022)
miR-214	Target AGO2	Induce ferroptosis in hepatocytes, promote liver injury	Luo et al. (2023b)
miR-125a	Target NEMO	Induce ferroptosis in hepatocytes, promote liver injury	Tak et al. (2024)
miR-222	Target TFRC	Inhibit ferroptosis in HSCs, promote liver fibrosis	Zhang et al. (2023b)
miR-15a	Interact with Gα12	Inhibit ferroptosis in hepatocytes, Inhibit liver fibrosis	Tak et al. (2022)
miR-124-3p	Target STEAP3	Inhibit ferroptosis in hepatocyte, inhibit liver injury	Wu et al. (2022b)
miR-6945-3p	Target DNMT3B	Induce ferroptosis in HSCs, inhibit liver fibrosis	Hu et al. (2024)
miR-26a	Downregulate SLC7A11	Induce ferroptosis in HSCs, inhibit liver fibrosis	Cao et al. (2024)
miR-142-3p	Target M1 macrophages	Induce ferroptosis in M1 macrophages, Inhibit liver fibrosis	Hu et al. (2022), Ma et al. (2017)
LINC00207	Target microRNA-761/HIGD1A	Inhibit ferroptosis in hepatocytes, inhibit liver injury	Li et al. (2024b)
LncRNA ZFAS1	Target RACK1/AMPKα	Induce ferroptosis in hepatocytes, promote liver injury	Yang et al. (2024b)
lncRNA MALAT1	Target miR-485-5p/MUC1	Induce ferroptosis in hepatocytes, promote liver fibrosis	Zhang et al. (2023c)
lncRNA FRMD6-AS1	Target miR-491-5p/USP13	Inhibit ferroptosis in HSCs , promote liver fibrosis	Li et al. (2024a)
TUG1	CYBB-hsa-miR-196a/b-5p-TUG1-PDK4	Inhibit ferroptosis in HSCs, promote liver fibrosis	Dong et al. (2023), Zhang et al. (2023d)
circFBXW4	Target miR-18b-3p/FBXW7	Induce ferroptosis in HSCs, inhibit liver fibrosis	Chen et al. (2020), Zhang et al. (2020)
CircDCBLD2	Interact with HuR	Induce ferroptosis in HSCs, inhibit liver fibrosis	Wang et al. (2024a)
circ-ITCH	Target Nrf2	Inhibit ferroptosis in hepatocytes, inhibit liver injury	Yang et al. (2022)

### 4.1 Ferroptosis-related miRNA and liver fibrosis

miRNA, a class of ncRNAs comprising 18–25 nucleotides, regulate protein translation by binding to the 3′untranslated regions (3′UTRs) of mRNAs, consequently inhibiting or inducing ferroptosis (Tabnak et al., 2021). In recent years, numerous studies have reported that miRNAs can mediate ferroptosis-related genes to regulate ferroptosis, thereby promoting or reversing liver fibrosis. miRNAs mediate ferroptosis to promote liver fibrosis. For example, in both *in vivo* and *in vitro* models of alcoholic liver disease (ALD), the overexpression of miR-192-5p markedly reduced the expression of fibronectin type III domain-containing protein 3B (FNDC3B) in hepatocytes. The depletion of FNDC3B markedly exacerbated ethanol-induced lipid peroxidation and iron overload, modulating ferroptosis to further exacerbate liver injury. Administering the ferroptosis inhibitor ferrostatin-1 (Fer-1) diminished FNDC3B inhibition-induced lipid peroxidation and ameliorated alcohol-induced liver injury (You et al., 2022). Luo et al. established an ALD model and found that miR-214 selectively binds to AGO2 protein in the nucleus, activating ferroptosis-driving genes (ACSL4, PRKAA2, and SLC38A1) to enhance ferroptosis in hepatocytes and exacerbate alcoholic liver injury (Luo J. et al., 2023). ALI has been shown to trigger ER stress-induced overexpression of Gα12, elevating miR-125a levels, suppressing NEMO expression, and downregulating GPX4, thereby intensifying hepatocellular ferroptosis (Tak et al., 2024). It has been reported that miR-15a is a downstream regulator of the Gα12 pathway in normal hepatocytes, directly interacting with the 3′-UTR of ALOX12 mRNA, thereby suppressing ALOX12 expression. In mice exposed to excessive acetaminophen (APAP) or in patients with acute liver injury or fibrosis, endoplasmic reticulum (ER) stress induces Gα12 overexpression, resulting in the downregulation of miR-15a levels. This leads to enhanced ALOX12 expression, which, through the accumulation of lipid hydroperoxides, depletes GSH and inhibits GPX4, thereby promoting ferroptosis in hepatocytes and exacerbating liver damage and fibrosis progression (Tak et al., 2022). Increased miR-222 expression in the serum of HCV-infected patients is particularly significant in both early and late stages of liver fibrosis, serving as a biomarker for detecting liver fibrosis (Abdel-Al et al., 2018). Zhang et al. found that TFRC is a downstream target of miR-222. Exosomal miR-222 from HBV-infected hepatocytes induces ferroptosis in LX-2 cells by inhibiting TFRC, ultimately aggravating liver fibrosis (Zhang Q. et al., 2023).

On the other hand, miRNAs can mediate ferroptosis to alleviate liver fibrosis. Hu et al. discovered that miR-142-3p expression is significantly enhanced in the peripheral blood of HBV-positive HCC patients, which can induce ferroptosis in M1 macrophages (Hu et al., 2022). Suppressing M1 macrophages decreases TNF-α levels, thereby mitigating liver fibrosis (Sheng et al., 2020). Cao et al. identified SLC7A11 as a downstream target of miR-26a through TargetScan and miRDB. In both *in vivo* and *in vitro* studies, mesenchymal stem cell-derived exosomal miR-26a was shown to inhibit HSCs activation by downregulating SLC7A11 expression, thereby promoting ferroptosis in HSCs and mitigating liver fibrosis (Cao et al., 2024). G-Rg3 induces ferroptosis in HSCs and mitigates liver fibrosis by reducing ACSL4 methylation via miR-6945-3p-mediated inhibition of DNMT3B (106). Steap3 is a reductase that converts ferric iron to ferrous iron, regulating intracellular iron homeostasis (Liu J. et al., 2021). A study found a binding site for miR-124-3p in Steap3. Using a rat liver transplantation model and hepatocyte hypoxia and reoxygenation (H/R) model, they injected heme oxygenase-1 (HO-1) modified bone marrow mesenchymal stem cell (BMMSC)-derived exosomes (HM-exos). These HM-exos, rich in miR-124-3p, inhibited STEAP3, reducing ferroptosis in H/R-treated cells. This significantly decreased neutrophil infiltration and levels of inflammatory factors in liver tissue, thereby suppressing the inflammatory response and mitigating liver damage (Wu L. et al., 2022).

### 4.2 Ferroptosis-related lncRNA and liver fibrosis

lncRNA, exceeding 200 nucleotides in length, have crucial roles in various pathways. They influence DNA replication, chromatin organization, gene transcription, and nascent RNA modification in the nucleus, while in the cytoplasm, they regulate mRNA stability and translation, as well as coordinate protein stability and function (Herman et al., 2022). Similar to miRNAs, lncRNA affect gene expression through various mechanisms. As competitive endogenous RNAs, they interact with miRNAs, targeting specific mRNA regions and interfering with gene expression (Shan et al., 2023).lncRNA can function as potential biomarkers for the prognosis and progression of liver fibrosis as well as direct therapeutic targets. Li et al. found that lncRNA FRMD6-AS1 inhibits HSC ferroptosis through negative regulation of the miR-491-5p/USP13 pathway in both *in vitro* and *in vivo* models, promoting ECM deposition and advancing liver fibrosis. Knockdown of FRMD6-AS1 significantly increased iron ion, ROS, and MDA levels in activated HSCs while reducing GSH levels and the expression of SLC7A11 and GPX4 proteins, inducing ferroptosis in HSCs, thereby restoring liver tissue structure and reversing liver fibrosis (Li Z. et al., 2024). In patients with chronic hepatitis B (CHB) combined with NAFLD, hypoxia inducible gene domain family member 1A (HIGD1A) was significantly upregulated. Further studies showed that LINC00207 upregulated HIGD1A via the microRNA-761-HIGD1A axis, activating GSH, reducing hepatic ROS levels, and alleviating oxidative stress-induced hepatocyte damage (Zhang H. et al., 2023).Recent research compared serum lncRNA levels between NAFLD patients and healthy controls, revealing significantly elevated lncRNA MALAT1 levels in NAFLD patients. MALAT1 targets the miR-485-5p-MUC1 axis in NAFLD to regulate ferroptosis in hepatocytes and promote the progression of liver fibrosis (Dong et al., 2023). In hepatic ischemia-reperfusion injury, the receptor for activated C kinase 1 (RACK1) directly interacts with AMPKα to regulate its phosphorylation at the Thr172 site, thereby modulating system Xc− activity. This process suppresses lipid peroxidation and ROS accumulation while enhancing glutathione levels, ultimately shielding hepatocytes from ferroptosis triggered by IRI. Moreover, studies have demonstrated that the lncRNA ZFAS1 exacerbates hepatocyte ferroptosis by inhibiting the RACK1/AMPKα signaling pathway (Yang Z. et al., 2024). Dong et al. analyzed gene samples using bioinformatics and identified CYBB as a co-regulated gene in NAFLD, leading to oxidative DNA damage. The taurine upregulated gene 1 (TUG1), a 7.1 kb long lncRNA, may modulate ferroptosis via the CYBB-hsa-miR-196a/b-5p-TUG1 axis, thereby affecting the progression of liver disease (Zhang X. et al., 2023). Additionally, some studies have focused on TUG1’s role in energy metabolism and ferroptosis regulation in liver fibrosis. Zhang et al. indicated that TUG1 is overexpressed in fibrotic liver tissues and HSCs, and it exacerbates liver fibrosis by reducing ferroptosis through enhanced glycolytic metabolism mediated by pyruvate dehydrogenase kinase isozyme 4 (PDK4) (Li MR. et al., 2024). These studies indicate that the differential expression of ferroptosis-related lncRNA in liver fibrosis offers clinical insights for the diagnosis, treatment, and prognosis of this condition.

### 4.3 Ferroptosis-related circRNA and liver fibrosis

circRNAs are RNA molecules with a unique circular structure, lacking 5′end caps and 3′end poly A tails, and are structurally more stable than linear RNAs (Li, 2023). They regulate transcription and RNA splicing, bind to mRNAs to influence gene expression, affect the stability and translation of cytoplasmic mRNAs, and interfere with signal transduction (Liu and Chen, 2022).With the advancement of technology and research methods, the regulatory mechanisms of circRNAs in liver fibrosis progression have been increasingly studied. Numerous circRNAs have been identified as significant regulators of liver fibrosis development. Chen et al. used circRNA sequencing (circRNA-seq) to obtain the circRNA expression profile in mouse HSCs, discovering that circFBXW4 was significantly downregulated in liver fibrosis. Overexpression of circFBXW4 inhibited HSC activation and proliferation, reduced collagen deposition, alleviated liver fibrosis progression in mice, and exhibited anti-inflammatory effects. Mechanistically, circFBXW4 inhibited HSC activation and alleviated HF through the miR-18b-3p/FBXW7 axis (Chen et al., 2020). Studies have shown that its downstream target, the ubiquitin ligase FBXW7, exhibited increased binding to RNA-binding protein ZFP36 in mice treated with the ferroptosis inducers erastin and sorafenib. Downregulation of ZFP36 in HSCs activated ferritinophagy, promoted HSC ferroptosis, and countered liver fibrosis (Zhang et al., 2020). HuR, a key RNA-binding protein (RBP), interacts with circDCBLD2 to enhance STUB1 expression and facilitate PARK7 ubiquitination, thereby inducing ferroptosis in HSCs and mitigating liver fibrosis (Wang J. et al., 2024). In a diabetic rat model of liver oxidative damage, upregulation of circ-ITCH was found to promote the Nrf2 signaling pathway, reducing cell oxidative damage caused by ROS (Fu et al., 2024). Upon external stimulation, Nrf2 translocates to the hepatocyte nucleus and activates downstream molecules such as heme oxygenase-1 (HO-1), inhibiting ROS production and thereby preventing ferroptosis and liver injury (Yang et al., 2022).To date, the role of ferroptosis-related circRNA, particularly its association with liver fibrosis, has not been fully elucidated. Therefore, further research is necessary to identify novel therapeutic targets for liver fibrosis.

While ncRNAs exhibit considerable potential in treating liver fibrosis, it is crucial to recognize that research targeting ncRNAs as future therapeutic strategies continues to face numerous challenges. Firstly, despite the growing comprehensiveness of ncRNA-related databases, specialized and integrative resources dedicated to ferroptosis-related ncRNAs in liver fibrosis are still lacking. This scarcity hampers the validation of ferroptosis-related ncRNAs, subsequently restricting the progress and depth of research in this domain and slowing the field’s advancement. Currently, the majority of studies are still in their early stages and predominantly rely on cell and animal models, which partially limits the physiological relevance of the findings. Using the *in vitro* stimulation model of LX-2 cells as an example, while this model serves as a foundational tool for basic research, its artificially constructed environment cannot fully recapitulate the intricate mechanisms of liver fibrosis *in vivo*, thus constraining the translational relevance of the findings to clinical practice. Furthermore, within the intricate microenvironment of liver fibrosis, ncRNAs are susceptible to degradation and exhibit poor stability, leading to low *in vivo* utilization efficiency. Consequently, devising strategies to effectively and precisely deliver ncRNAs to specific liver cell types, such as HSCs or hepatocytes, while preserving their stability *in vivo* remains a pivotal technical challenge. In conclusion, extensive basic research and well-structured clinical trials are indispensable for promoting the clinical application of emerging ncRNA-based therapies in liver fibrosis treatment. Such efforts are directed toward addressing current limitations and facilitating more efficient therapeutic translation.

## 5 Advances in the treatment of liver fibrosis

With the advancing understanding of liver fibrosis pathogenesis, numerous therapeutic strategies have emerged. Beyond targeting ferroptosis-related ncRNA therapies, this discussion will also briefly review other contemporary approaches for treating liver fibrosis, such as molecular targeted therapy, immunotherapy, gut microbiota modulation and nanomedicine. These strategies underscore the multifaceted research trajectories explored by scholars in this domain.

Molecular targeted therapy primarily aims to modulate signaling pathways, cytokines, and molecules implicated in the fibrosis process, thereby slowing or potentially reversing fibrosis progression. Activation of the TGF-β1/Smad signaling pathway is a crucial step in promoting liver fibrosis. It triggers HSCs activation, stimulates excessive extracellular matrix production and deposition, thus accelerating fibrosis progression. Fluorofenidone suppresses the TGF-β1/Smad signaling pathway through upregulation of Smad7 expression in hepatic stellate cells (HSCs), thereby preventing excessive HSC activation in liver fibrosis mice models and attenuating fibrosis progression (Peng et al., 2019). Activation of Notch signaling downregulates endothelial nitric oxide synthase (eNOS)/soluble guanylate cyclase (sGC) signaling, thereby inducing LSEC dedifferentiation and exacerbating liver fibrosis. However, the application of sGC activators reverses LSEC dedifferentiation and ameliorates liver fibrosis (Duan et al., 2018). Nevertheless, the pathogenesis of liver fibrosis is highly intricate, encompassing multiple signaling pathways and molecular mechanisms. Consequently, single-target therapies are unlikely to effectively address the diverse pathological processes involved.

The liver possesses a complex immune microenvironment that undergoes substantial changes during liver injury. Hepatocytes release abundant chemokines and cytokines, gradually reshaping the immune microenvironment. As a result, diverse immune cells are recruited to the injury sites, exerting pivotal roles in the progression of liver fibrosis. During liver injury, activated HSCs secrete inflammatory chemokines, such as CCL2 and CCL4, to recruit macrophages to the site of injury. These macrophages subsequently release inflammatory cytokines that perpetuate HSC activation (Weiskirchen and Tacke, 2014). Cenicriviroc (CVC), an innovative oral inhibitor targeting chemokine receptors 2 and 5, exhibited efficacy in reducing fibrosis by attenuating the recruitment of inflammatory macrophages in the CENTAUR phase IIb trial. Nevertheless, the AURORA phase III study revealed no significant improvement in liver fibrosis after 12 months of treatment (Anstee et al., 2024). During the early stages of liver fibrosis, NK cells release substantial amounts of perforin and granzyme, inducing apoptosis in activated HSCs. However, as fibrosis advances, HSCs secrete excessive TGF-β, which suppresses NK cell production of cytotoxic substances and cytokines. Integrating NK cell-based therapies with TGF-β inhibitors could offer a promising antifibrotic treatment strategy (Zhang Y. et al., 2022). T-cell immunotherapy for chronic hepatitis B (CHB) employing TCR-redirected T cells aims to mitigate the progression of HBV-associated liver fibrosis (Tan and Schreiber, 2020). Prolonged use of immune therapy may result in immune tolerance or immune evasion and pose potential systemic side effects, such as hyperactive immune responses, off-target effects, and liver tissue injury.

The gut microbiota plays a central regulatory role in the initiation and progression of liver fibrosis. Recent studies underscore the pivotal role of the gut-liver axis in a range of liver diseases, including liver fibrosis. Specifically, bacterial translocation products (e.g., LPS, peptidoglycan, or live bacteria) enter the liver through the portal circulation and contribute to the fibrosis process by modulating the hepatic immune microenvironment via TLRs (Albillos et al., 2020). Treatment of KK-Ay mice with pioglitazone led to an increased proportion of lactobacilli in the gut microbiota, accompanied by elevated levels of the LA metabolite HYA (10-hydroxy-cis-12-octadecenoic acid), produced by lactobacilli. Moreover, HYA was shown to inhibit the TGF-β-Smad3 signaling pathway, thereby attenuating the progression of NAFLD fibrosis (Kasahara et al., 2023). Fecal microbiota transplantation of human NAFLD gut microbiota into recipient mice has been reported to enhance hepatic inflammatory B cell activation via the TLR4-MyD88 and BCR signaling pathways, thereby promoting liver fibrosis (Barrow et al., 2021). Modulating the gut microbiota presents a promising therapeutic strategy; however, due to its dynamic and intricate nature, precise regulation remains a significant challenge. Further studies are required to elucidate its mechanisms in liver diseases and promote its clinical application.

Nanomedicine integrates nanoparticle technology with the field of medicine, employing nanoparticles to encapsulate and deliver drugs, or utilizing nanoparticles as therapeutic agents themselves. This strategy facilitates the targeted delivery of drugs, genes, or other therapeutic compounds to the disease site, thereby enhancing therapeutic efficacy while minimizing side effects (Zhang P. et al., 2023). Wang et al. developed a nanoparticle system based on silica cross-linked micelles (SCLMs), modified with the peptide CTCE9908 (CT-SCLMs), which selectively binds to the CXCR4 receptor, highly expressed on activated HSCs. The CT-SCLMs, loaded with two anti-fibrosis drugs, silibinin and sorafenib, effectively inhibit the proliferation of activated HSCs, alleviate liver inflammation, and reduce collagen deposition (Wang L. et al., 2024). Graphene-Dendrimer Nanostars were utilized to deliver plasmids encoding collagenase MMP-9 into inflammatory macrophages, resulting in the overexpression of MMP-9. This process also facilitated the transition from inflammatory M1 to anti-inflammatory M2 macrophages, selectively attenuating liver fibrosis (Melgar-Lesmes et al., 2018). Nanomedicine has demonstrated significant potential in the treatment of liver fibrosis, yet it faces distinct challenges. The dense fibrous structures characteristic of liver fibrosis hinder the deep penetration of nanoparticles within the liver. Furthermore, the accumulation of KCs and other immune cells during the progression of liver fibrosis can result in the rapid clearance of certain nanodrugs. Therefore, advancements in more efficient and safer nanomaterials, along with precise delivery technologies, are essential (Ezhilarasan, 2021).

## 6 Discussion

Liver diseases, encompassing liver injury, NAFLD, ALD, liver cirrhosis, and liver cancer, are increasingly contributing to the global disease burden, presenting significant threats to human health and life (Terrault et al., 2023). Liver fibrosis often presents without discernible clinical symptoms; however, as it progresses, the liver undergoes a range of cellular transformations. These include hepatocyte apoptosis and necrosis, secretion of inflammatory mediators, recruitment of immune cells, and activation of HSCs (Hammerich and Tacke, 2023).Persistent liver fibrosis is recognized as a crucial factor in the further advancement of various liver diseases, significantly impacting the prognosis of chronic liver conditions. This underscores the importance of early intervention in liver fibrosis to halt disease progression. Epigenetic modifications of ncRNAs, particularly miRNA, lncRNA, and circRNA, have been demonstrated to regulate ferroptosis mechanisms across various pathological conditions (Zheng and Zhang, 2023). As both agonists and inhibitors, ncRNAs modulate ferroptosis pathways, revealing a complex regulatory network. Ferroptosis-related ncRNAs play a key role in the development of liver fibrosis, emerging as significant pathways for the treatment and prevention of liver fibrosis. Notably, some ferroptosis-related ncRNAs have been implicated in cellular energy metabolism within liver fibrosis, where their dysregulation may be pivotal in the onset and progression of the disease. However, the detailed mechanisms linking ferroptosis, ferroptosis-related ncRNAs, and liver fibrosis remain unclear. In recent years, there has been rapid progress in understanding the relationships between ferroptosis, ferroptosis-related ncRNAs, and liver fibrosis. These findings conclude that ferroptosis and its related ncRNAs are pivotal in the onset and prognosis of liver fibrosis, holding promising potential for future therapeutic and prognostic developments. Nonetheless, given the involvement of various ncRNAs in the progression of liver fibrosis through ferroptosis pathways, it remains uncertain which specific ferroptosis-related ncRNAs determine the pathogenesis or stages of liver fibrosis. Many challenges still need to be addressed in future research. These findings conclude that ferroptosis and its associated ncRNAs are pivotal in the onset and prognosis of liver fibrosis.
